# Video laryngoscopy versus direct laryngoscopy for first-attempt tracheal intubation in the general ward

**DOI:** 10.1186/s13613-018-0428-0

**Published:** 2018-08-13

**Authors:** Moon Seong Baek, MyongJa Han, Jin Won Huh, Chae-Man Lim, Younsuck Koh, Sang-Bum Hong

**Affiliations:** 1 Department of Pulmonary and Critical Care Medicine, Asan Medical Centre, University of Ulsan College of Medicine, 88 Olympic-ro 43-gil, Songpa-gu, Seoul, 05505 Republic of Korea; 2 Medical Emergency Team, Asan Medical Centre, University of Ulsan College of Medicine, Seoul, Republic of Korea

**Keywords:** Laryngoscopy, Intubation, Critical illness

## Abstract

**Background:**

Recent trials showed that video laryngoscopy (VL) did not yield higher first-attempt tracheal intubation success rate than direct laryngoscopy (DL) and was associated with higher rates of complications. Tracheal intubation can be more challenging in the general ward than in the intensive care unit. This study aimed to investigate which laryngoscopy mode is associated with higher first-attempt intubation success in a general ward.

**Methods:**

This is a retrospective study of tracheal intubations conducted at a tertiary academic hospital. This analysis included all intubations performed by the medical emergency team in the general ward during a 48-month period.

**Results:**

For the 958 included patients, the initial laryngoscopy mode was video laryngoscopy in 493 (52%) and direct laryngoscopy in 465 patients (48%). The overall first-attempt success rate was 69% (664 patients). The first-attempt success rate was higher with VL (79%; 391/493) than with DL (59%; 273/465, *p* < 0.001). The first-attempt intubation success rate was higher among experienced operators (83%; 266/319) than among inexperienced operators (62%; 398/639, *p* < 0.001). In multivariate logistic regression analyses, VL, pre-intubation heart rate, pre-intubation SpO_2_ > 80%, a non-predicted difficult airway, experienced operator, and Cormack–Lehane grade were associated with first-attempt intubation success in the general ward. Over all intubation-related complications were not different between two groups (27% for VL vs. 25% for DL). However, incidence of a post-intubation SpO_2_ < 80% was higher with VL than with DL (4% vs. 1%, *p* = 0.005), and in-hospital mortality was also higher (53.8% vs. 43%, *p* = 0.001).

**Conclusion:**

In a general ward setting, the first-attempt intubation success rate was higher with video laryngoscopy than with direct laryngoscopy. However, video laryngoscopy did not reduce intubation-related complications. Furthers trials on best way to perform intubation in the emergency settings are required.

**Electronic supplementary material:**

The online version of this article (10.1186/s13613-018-0428-0) contains supplementary material, which is available to authorized users.

## Background

Tracheal intubation can be a hazardous procedure outside of the operating room, as it tends to be performed by inexperienced junior trainees and involves physiologically unstable patients. Accordingly, tracheal intubation outside of the operating room is associated with a higher rate of complications than the corresponding procedure inside the operating room [1–3]. Furthermore, a successful first-attempt intubation is important in the emergency setting [4] because related complications are associated with multiple intubation attempts [5, 6].

A video laryngoscope, defined as a laryngoscopic device to which a camera has been attached to the tip of the blade, could assist airway management by improving visualisation of the glottis in critically ill patients [7–10]. According to a few studies, video laryngoscopy yields a greater rate of first-attempt intubation success than direct laryngoscopy [7, 11]. However, a recent randomised clinical trial in an intensive care unit (ICU) setting found that video laryngoscopy did not yield higher first-attempt tracheal intubation success rate than direct laryngoscopy and was associated with higher rates of complications [12]. Therefore, the superior device for first-attempt intubation success in critically ill patients remains controversial. Furthermore, limited data are available regarding tracheal intubation in general ward settings [5, 13].

The objective of this study was to investigate which laryngoscopy mode is associated with higher first-attempt intubation success in a general ward. We hypothesised that tracheal intubation with the video laryngoscope would be associated with increased successful intubation on first attempt compared with the direct laryngoscopy.

## Methods

### Study design and eligible patients

This retrospective study of tracheal intubations was conducted at Asan Medical Centre, a tertiary referral hospital in Korea. Tracheal intubation data between January 2012 and December 2015 were collected. The primary outcome was first-attempt success and secondary outcome was intubation-related complications. All patients aged ≥ 19 years who had been intubated in the ward by medical emergency team (MET) were eligible. Exclusion criteria were as follow: (1) tracheal intubations performed during cardiac arrest because the success of first-attempt intubation might have been affected by cardiopulmonary resuscitation, (2) patients who initially used supraglottic airway devices because the study aimed to compare the direct laryngoscopy and video laryngoscopy, (3) patients whose records were unavailable, 4) duplicated cases.

The institutional review board of the Asan Medical Centre approved this study (approval no. 2016-0599) and granted a waiver of patient consent because of the retrospective nature of the study.

### Study setting

The MET provides airway management to general ward patients who require immediate or cardiopulmonary resuscitation. A MET comprises attending critical care physicians, critical care medicine fellows, internal medicine residents, and critical care qualified nurses and is available 24 h per day, 7 days per week. If the MET is activated via a screening or calling system, the team members proceed to the intubation location with a portable airway bag. This bag contains a video laryngoscope, capnograph, i-gel (Intersurgical Ltd, Wokingham, Berkshire, UK), laryngeal tube (VBM Medizintechnik, Sulz, Germany), gum elastic bougie, tracheal tube exchanger (Cook airway exchange catheter), and percutaneous cricothyroidotomy kit (Melker Emergency Cricothyrotomy Kit, Cook Critical Care, Bloomington, Indiana). The laryngeal tube is one of the supraglottic airway devices that consists of an airway tube with a small cuff attached at the tip and a larger balloon cuff at the middle part of the tube. The i-gel is also a supraglottic airway device that features a non-inflatable cuff and the possibility to introduce a gastric catheter. Direct laryngoscopes were contained in the emergency trolley each general ward. Before starting rotations in ICU, medical residents received airway management programme, consisted with direct laryngoscopy and video laryngoscopy and i-gel, laryngeal tube. Airway management training programme for critical care medicine fellows is provided by attending physician once a month. The curriculum is designed with basic skills with direct laryngoscopy and video laryngoscopy and more difficult scenarios requiring execution with alternative techniques, including i-gel, laryngeal tube, gum elastic bougie, bronchoscopy, and cricothyroidotomy.

In this study, the intubation procedure was conducted in accordance with general guidelines for the airway management of critically ill patients [14, 15]. Although fentanyl (1–3 µg kg^−1^) and etomidate (0.3 mg kg^−1^) were the preferred pre-treatment and induction agents, the operators selected the sedatives and dosages after considering each patient’s condition. A sedative was administered 2 min after pre-treatment agent injection, and tracheal intubation was performed 20–30 s later. The operators chose either a curved Macintosh laryngoscope with metal reusable blade or a GlideScope video laryngoscope (Verathon, Bothell, WA, USA) as the initial device. Tracheal intubation was supervised by an experienced operator when performed by an inexperienced operator. If the second intubation attempt was unsuccessful, the experienced operator performed the third attempt. However, for the patient safety, supervisors tended to intubate directly if the patient was expected to have difficult airway or hemodynamically unstable. It is presumed that relatively less severe patients were intubated by inexperienced operators and with direct laryngoscopy for the training purpose. Correct tracheal tube placement was assessed using careful auscultation, end-tidal carbon dioxide measurement, and chest radiography. The end-tidal carbon dioxide level was measured using an EMMA Emergency Capnograph (Masimo Corp., Irvine, CA, USA). After each tracheal intubation, intubation-related information was recorded on a data collection sheet by MET nurses, and all tracheal intubations were reviewed in regular weekly meetings.

### Data collection and definition

The following information was recorded on the intubation data collection sheet: patient demographics, operator specialty, time and location of tracheal intubation, reason for intubation, number of intubation attempts, device(s) used, medications (pre-treatment agents, sedatives, and paralytics), complications during tracheal intubation, characteristics of the predicted difficult airway, Cormack–Lehane grade, and vital signs pre- and post-tracheal intubation.

We defined intubation duration as the time interval between infusion of the pre-treatment agent (or sedative if the patient did not receive a pre-treatment agent) and confirmation of tracheal tube placement by capnography. The board-certified physician or surgeon in the ICU (attending physician and fellow) was considered an experienced operator, while a medical or surgical resident-in-training was considered an inexperienced operator. A tracheal intubation attempt was defined as insertion of the laryngoscopy blade into the oral cavity, regardless of whether tracheal tube insertion was attempted. A first-attempt success was defined as the placement of a tracheal tube on the first attempt and difficult intubation was defined as more than two attempts of intubation [16].

Several factors were investigated before intubation to predict difficult airways. These factors included blood/vomitus/secretion in the airway, cervical immobilisation, neck trauma/mass or vocal cord palsy, the 3-3-2 rule, short neck, obesity, limited mouth opening, small mouth, and large tongue. The 3-3-2 rule was defined as an inter-incisor distance of < 3 fingers, a hyoid-mental distance of < 3 fingers, and a hyoid-thyroid cartilage distance of < 2 fingers.

An event occurring within 30 min after tracheal intubation was considered intubation-related complications. These events included hypotension (systolic arterial pressure < 90 mmHg despite adequate volume loading or inotrope use), severe desaturation (oxygen saturation < 80%), oesophageal intubation, dental injury (tooth extraction), oral bleeding, aspiration of gastric contents, and cardiac arrest/arrhythmia [17].

### Statistical analysis

Continuous variables are presented as median (interquartile range) or mean (standard deviation). Categorical variables are presented as number (percentage). Differences among categorised groups were compared using either the Chi-square test or Fisher’s exact test, and data for continuous variables were compared using the independent Student’s *t* test or Mann–Whitney *U* test. Univariate and multivariate logistic regressions using backward elimination method were performed to identify the factors associated with first-attempt intubation success and intubation-related complications. Calibration of the models was evaluated with the Hosmer–Lemeshow goodness-of-fit test. All statistical comparisons were two-sided, and a *p* value of < 0.05 was considered statistically significant. Data were analysed using the Statistical Package for the Social Sciences (SPSS), version 22.0 (IBM Corporation, Armonk, NY, USA). To reduce the effect of treatment-selection bias and potential confounding factors in an observational study, we performed an adjustment for differences in baseline characteristics of patients using a propensity-score matching (Additional file 1: Table S1). Compounding factors were age, sex, medical department, pre-intubation blood pressure, pre-intubation heart rate, pre-intubation oxygen saturation, predicted difficult airway, level of operator experience, pre-treatment agent, sedatives, and paralytic agents. Using these methods we could reduce or eliminate confounding by those measured covariates. A power analysis was performed with reference to similar studies conducted in an intensive care unit setting [7, 9, 18]. We determined the power of the study by assuming a first-pass success rate of 65% (direct laryngoscopy) and 80% (video laryngoscopy).

## Results

**Fig. 1:**
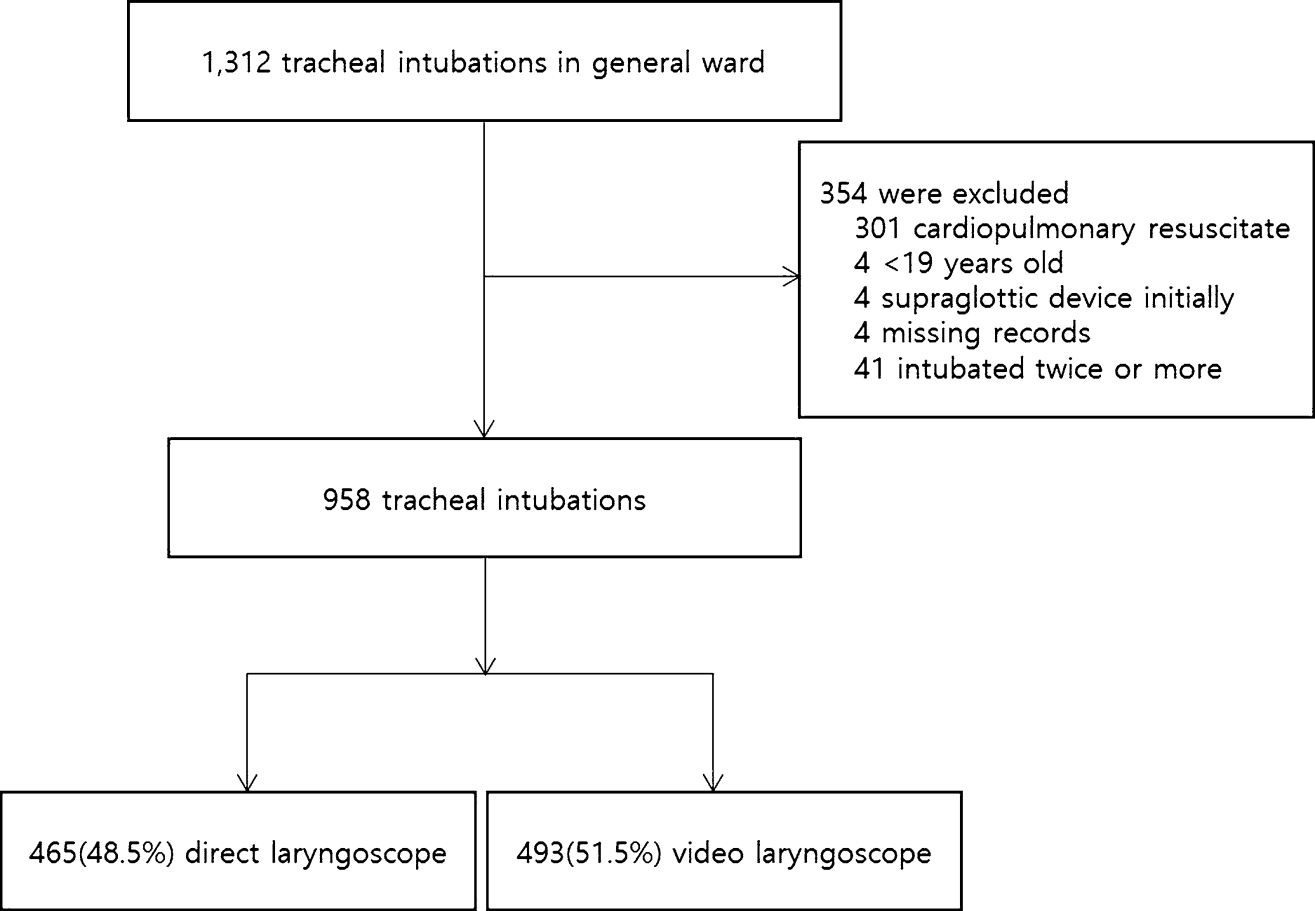
Flowchart of the study

During the study period, 1312 tracheal intubations were performed in general ward. A total of 354 patients were excluded which received tracheal intubation during cardiac arrest, 4 younger than 19 years, 4 which initially received tracheal intubation using a supraglottic airway device, 4 which records were unavailable. Because thirty-nine patients were intubated twice, and one patient was intubated three times, 41 cases also excluded. Among the 958 intubations, the initial laryngoscopy mode was video laryngoscopy in 493 (52%) and direct laryngoscopy in 465 (48%) (Fig. 1). As the alternative techniques, an i-gel and a laryngeal tube was used. Gum elastic bougie was used in four cases for 102 failures at first pass in the video laryngoscopy group and was not used for 192 failures in the direct laryngoscopy group. Tracheostomy was conducted in three patients. One tracheostomy was conducted after a failure of cricothyroidotomy. The other two tracheostomies were performed after failures of tracheal intubation because oxygen saturation was maintained at > 90%. One patient died after a failure of emergency cricothyroidotomy in a ‘cannot intubate, cannot oxygenate’ situation.

Hypoxic respiratory failure (59%) was the most common reason for intubation in the general ward. A predicted difficult airway was present in 202 intubations (20%), and this incidence was higher in the video laryngoscopy group (27%) than the direct laryngoscopy group (13%, *p* < 0.001; Table 1). The first intubation attempt was conducted by inexperienced operators in 67% of patients and by experienced operators in 33% of patients (Table 2). Video laryngoscopy was chosen by 62% (198/319) of experienced operators and by 46% (295/639, *p* < 0.001) of inexperienced operators. Overall, 20.7% of patients received NMB (Table 2).

**Table 1:** Baseline clinical characteristics of the patients according to intubation device

Variable	Total *n* = 958	Direct laryngoscopy *n* = 465	Video laryngoscopy *n* = 493	*p*
Age (years)	63 [53, 73]	66 [56, 74]	61 [51, 71]	< 0.001
Male, *n* (%)	621 (64.8)	318 (68.4)	303 (61.5)	0.025
Medical department*, *n* (%)	713 (74.4)	293 (63.0)	420 (85.2)	< 0.001
Reason for intubation, *n* (%)				
Airway protection	138 (14.4)	67 (14.4)	71 (14.4)	0.998
Hypercapnic respiratory failure	91 (9.5)	49 (10.5)	42 (8.5)	0.287
Hypoxic respiratory failure	567 (59.2)	273 (58.7)	294 (59.6)	0.771
Shock	44 (4.6)	22 (4.7)	22 (4.5)	0.843
Metabolic acidosis	84 (8.8)	33 (7.1)	51 (10.3)	0.076
Others	34 (3.5)	21 (4.5)	13 (2.6)	0.116
Pre-intubation				
Systolic blood pressure (mmHg)	125 ± 33	127 ± 34	122 ± 33	0.022
Diastolic blood pressure (mmHg)	72 ± 23	73 ± 22	71 ± 23	0.227
Heart rate (beats per minute)	120 ± 28	120 ± 28	121 ± 28	0.385
Oxygen saturation (%)	94 [88, 98]	94 [89, 98]	93 [88, 98]	0.003
Severe desaturation (SpO_2_ < 80%), *n* (%)	76 (8.6)	28 (6.5)	48 (10.6)	0.029
Pre-oxygenation devices, *n* (%)				< 0.001
Nasal cannula	154 (17.5)	79 (18.6)	75 (16.5)	
Venture mask	103 (11.7)	62 (14.6)	41 (9.0)	
Simple mask	14 (1.6)	7 (1.6)	7 (1.5)	
Reservoir mask	424 (48.2)	206 (48.5)	218 (47.9)	
High-flow nasal cannula	163 (18.5)	55 (12.9)	108 (23.7)	
BiPAP	22 (2.5)	16 (3.8)	6 (1.3)	
Predicted difficult airway, *n* (%)	194 (20.3)	59 (12.7)	135 (27.4)	< 0.001
Blood, vomitus, or secretion in airway	18 (1.9)	6 (1.3)	12 (2.4)	0.193
Cervical immobilisation	21 (2.2)	6 (1.3)	15 (3.0)	0.064
Neck trauma/mass or vocal cord palsy	47 (4.9)	15 (3.2)	32 (6.5)	0.019
Evaluate the 3-3-2 rule	37 (3.9)	7 (1.5)	30 (6.1)	< 0.001
Short neck	74 (7.7)	25 (5.4)	49 (9.9)	0.008
Obesity	32 (3.3)	9 (1.9)	23 (4.7)	0.019
Limited mouth opening/small mouth	59 (6.2)	15 (3.2)	44 (8.9)	< 0.001
Large tongue	16 (1.7)	8 (1.7)	8 (1.6)	0.906

Values are expressed as median (interquartile range), mean (standard deviation), or *n* (%)

*SpO*_*2*_ Oxygen saturation

* Departments were divided into two groups: medical and surgical departments

**Table 2:** Characteristics of the physicians and types of hypnotic medication and neuromuscular blocker

Variable	Total *n* = 958	Direct laryngoscopy *n* = 465	Video laryngoscopy *n* = 493	*p*
Level of operator experience*, n (%)				< 0.001
Inexperienced	639 (66.7)	344 (74.0)	295 (59.8)	
Experienced	319 (33.3)	121 (26.0)	198 (40.2)	
Pre-treatment agent, *n* (%)				
Fentanyl	722 (75.4)	335 (72.0)	387 (78.5)	0.021
Sedatives, *n* (%)	877 (91.5)	427 (91.8)	450 (91.3)	0.760
Etomidate	814 (85.0)	384 (82.6)	430 (87.2)	0.045
Ketamine	24 (2.5)	12 (2.6)	12 (2.4)	0.885
Midazolam	58 (6.1)	49 (10.5)	9 (1.8)	< 0.001
Other^†^	10 (1.0)	4 (0.9)	6 (1.2)	0.754
Paralytic agents, *n* (%)	198 (20.7)	125 (26.9)	73 (14.8)	< 0.001
Succinylcholine	150 (15.7)	106 (22.8)	44 (8.9)	< 0.001
Rocuronium	9 (0.9)	5 (1.1)	4 (0.8)	0.746
Other^‡^	41 (4.3)	15 (3.2)	26 (5.3)	0.118

Values are expressed as *n* (%)

*IM* internal medicine

* Operators were divided into two groups by the level of experience at the first intubation attempt. An experienced operator was defined as a board-certified physician or surgeon working in a critical care unit; an inexperienced operator was defined as a medical or surgical resident-in-training

^†^Included propofol and Ativan

^‡^Included atracurium and cisatracurium

**Fig. 2:**
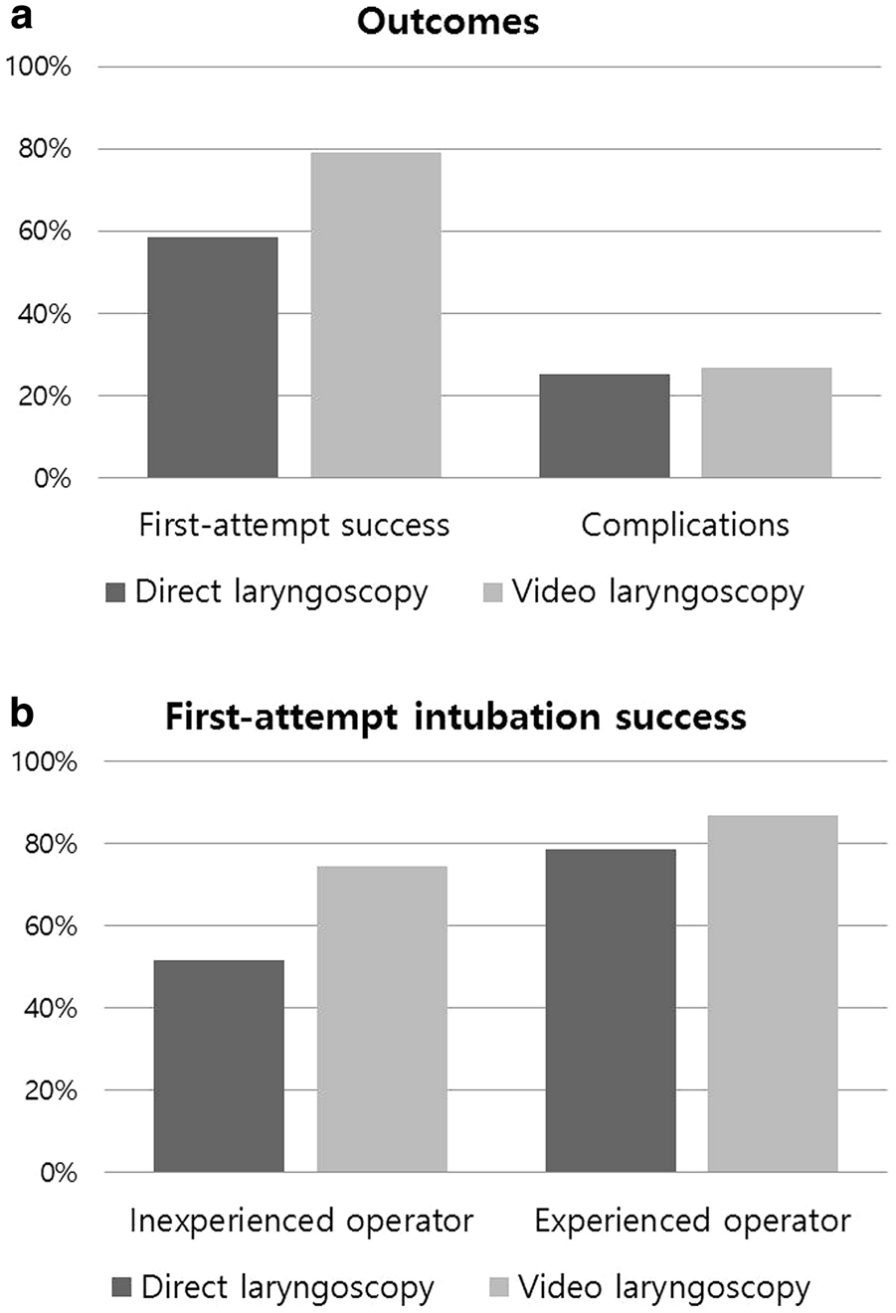
Main results of the study. **a** Outcomes, **b** first-attempt intubation success

The overall first-attempt intubation success rate was 69%, with rates of 79% and 59% in the video laryngoscopy and direct laryngoscopy groups, respectively (Fig. 2). Among 192 unsuccessful tracheal intubation with direct laryngoscopy, second intubation attempt was performed by 90 (47%) video laryngoscopy. On the contrary, 102 unsuccessful tracheal intubation with video laryngoscopy changed to 7 (7%) direct laryngoscopy. Among inexperienced operators, the first-attempt intubation success rate was higher with video laryngoscopy (75%) than with direct laryngoscopy (52%, *p* < 0.001; Table 7). The incidence of difficult intubation was 294 (31%). The incidence of difficult intubation was higher in the direct laryngoscopy group (41%) than in the video laryngoscopy group (21%, *p* < 0.001; Table 3).

**Table 3:** Outcomes according to intubation device

Variable	Total *n* = 958	Direct laryngoscopy *n* = 465	Video laryngoscopy *n* = 493	*p*
First-attempt success, *n* (%)	664 (69.3)	273 (58.7)	391 (79.3)	< 0.001
Difficult intubation*, *n* (%)	294 (30.7)	192 (41.3)	102 (20.7)	< 0.001
Cormack–Lehane grade^†^, *n* (%)				0.024
1	721 (76.8)	328 (72.6)	393 (80.7)	
2	170 (18.1)	95 (21.0)	75 (15.4)	
3	38 (4.0)	22 (4.9)	16 (3.3)	
4	10 (1.1)	7 (1.5)	3 (0.6)	
No. of intubation attempts	1 [1, 2]	1 [1, 2]	1 [1, 1]	< 0.001
Intubation duration^‡^ (min)	4 [3, 6)	4 [3, 7]	4 [3, 5]	< 0.001

Values are expressed as median (interquartile range) or *n* (%)

* Defined as more than two attempts of intubation

^†^Reflects glottis visualisation, with a score range of 1 (good) to 4 (no glottis visualisation)

^‡^Defined as the time between the infusion of induction medication and confirmation of endotracheal tube placement by capnography or chest radiography

A Cormack–Lehane grade of 3 or 4 (poor glottis visualisation) was more frequent in the direct laryngoscopy group (6% vs. 4%, *p* = 0.024). The number of intubation attempts was fewer with video laryngoscopy than with direct laryngoscopy, with an absolute difference of 0.32 (95% CI: 0.21–0.42, *p* < 0.001). The intubation duration was also shorter with video laryngoscopy than with direct laryngoscopy, with an absolute difference of 0.74 min (95% CI: 0.31–1.20, *p* = 0.001). No significant differences were observed in intubation-related complications according to the device used (27% in the video laryngoscopy group vs. 25% in the direct laryngoscopy group, *p* = 0.573; Table 4). The incidence of a pre-intubation SpO_2_ < 80% was higher in the video laryngoscopy group (11% vs. direct laryngoscopy group, 7%; *p* = 0.029); similarly, the incidence of a post-intubation SpO_2_ < 80% was also higher with video laryngoscopy (4% vs. 1%, *p* = 0.005; Table 4).

**Table 4:** Complications according to intubation device

Variable	Total *n* = 958	Direct laryngoscopy *n* = 465	Video laryngoscopy *n* = 493	*p*
Complications, *n* (%)	251 (26.2)	118 (25.4)	133 (27.0)	0.573
Hypotension*	181 (18.9)	94 (20.2)	87 (17.6)	0.310
Severe desaturation^†^	24 (2.6)	5 (1.1)	19 (4.1)	0.005
Oesophageal intubation	1 (0.1)	1 (0.2)	0 (0.0)	0.485
Dental trauma	4 (0.4)	1 (0.2)	3 (0.6)	0.625
Oral bleeding	42 (4.4)	18 (3.9)	24 (4.9)	0.451
Aspiration	3 (0.3)	2 (0.4)	1 (0.2)	0.614
Others^‡^	12 (1.3)	3 (0.6)	9 (1.8)	0.101
Length of hospital stay (days)	32 [17, 58]	32 [18, 58]	31 [16, 59]	0.640
In-hospital mortality (%)	465 (48.5)	200 (43.0)	265 (53.8)	0.001

* Defined as a systolic blood pressure < 90 mmHg

^†^Defined as an oxygen saturation < 80%

^‡^Included cardiopulmonary resuscitation or bradycardia

In multivariate logistic regression analyses, video laryngoscopy (odds ratio, 95% CI 3.058, 2.192–4.267, *p* < 0.001), pre-intubation heart rate (1.006, 1.000–1.011, *p* = 0.044), pre-intubation SpO_2_ < 80% (0.521, 0.316–0.861, *p* = 0.011), a predicted difficult airway (0.344, 0.230–0.514, *p* < 0.001), experienced operator (3.319, 2.284–4.823, *p* < 0.001), and Cormack–Lehane grade (0.459, 0.352–0.599, *p* < 0.001) were associated with first-attempt intubation success in the general ward (Table 5). The use of sedatives (OR, 95% CI: 2.377, 1.224–4.616, *p* = 0.011), paralytic agents (OR, 95% CI: 0.449, 0.293–0.687, *p* < 0.001), and the number of intubation attempts (OR, 95% CI: 1.535, 1.291–1.825, *p* < 0.001) were associated with intubation-related complications (Table 6). In the propensity-score matching, video laryngoscopy group has increased odds of first-attempt intubation success (OR, 95% CI: 2.450, 1.696, 3.539, *p* < 0.001; Additional file 2: Table S2). After propensity-score matching, the first-attempt success rate was 60% (181/300) in direct laryngoscopy group, and 80% (239/300) in video laryngoscopy group. The procedure-related complication rate showed 27% in direct laryngoscopy group, and 26% in video laryngoscopy group. Table 7 demonstrated that subgroup analysis of the outcomes. In the inexperienced operators, video laryngoscopy group had higher first-attempt success rate than direct laryngoscopy group. On the other hand, there was no significant difference on the intubation-related complications in the experienced operators. In the patients without predicted difficult airway, video laryngoscopy group had greater first-intubation success rate than direct laryngoscopy group.

**Table 5:** Factors associated with first-attempt intubation success

Variable	Univariate analysis	*p*	Multivariate analysis	*p*
	OR (95% CI)		OR (95% CI)	
Age	0.992 (0.982, 1.002)	0.102		
Female	1.031 (0.773, 1.375)	0.835		
Video laryngoscopy	2.696 (2.026, 3.587)	< 0.001	3.058 (2.192, 4.267)	< 0.001
Medical department	1.346 (0.990, 1.831)	0.058		
Systolic blood pressure (mmHg)	0.998 (0.994, 1.002)	0.380		
Diastolic blood pressure (mmHg)	1.000 (0.994, 1.006)	0.931		
Heart rate (beats per minute)	1.006 (1.001, 1.011)	0.021	1.006 (1.000, 1.011)	0.044
Oxygen saturation (%)	1.012 (0.999, 1.025)	0.076		
Severe desaturation (SpO_2_ < 80%), *n* (%)	0.559 (0.347, 0.901)	0.017	0.521 (0.316, 0.861)	0.011
Predicted difficult airway	0.388 (0.280, 0.536)	< 0.001	0.344 (0.230, 0.514)	< 0.001
Experienced operator	3.039 (2.173, 4.250)	< 0.001	3.319 (2.284, 4.823)	< 0.001
Pre-treatment agent	1.127 (0.822, 1.545)	0.457		
Sedatives	0.722 (0.427, 1.220)	0.223		
Paralytic agents	0.857 (0.614, 1.197)	0.365		
Cormack–Lehane grade	0.384 (0.301, 0.489)	< 0.001	0.459 (0.352, 0.599)	< 0.001

*OR* odds ratio, *CI* confidence interval

**Table 6:** Factors associated with intubation-related complications

Variables	Univariate analysis	*p*	Multivariate analysis	*p*
	OR (95% CI)		OR (95% CI)	
Age	1.006 (0.996, 1.017)	0.250		
Female	0.993 (0.734, 1.343)	0.964		
Video laryngoscopy	1.086 (0.814, 1.450)	0.573	1.173 (0.865, 1.591)	0.306
Medical department	1.393 (0.986, 1.968)	0.060		
Systolic blood pressure (mmHg)	0.998 (0.994, 1.002)	0.366		
Diastolic blood pressure (mmHg)	0.995 (0.989, 1.002)	0.165		
Heart rate (beats per minute)	0.999 (0.994, 1.004)	0.747		
Oxygen saturation (%)	0.990 (0.977, 1.003)	0.134		
Severe desaturation (SpO_2_ < 80%), *n* (%)	1.408 (0.851, 2.329)	0.183		
Predicted difficult airway	1.302 (0.920, 1.842)	0.136		
Experienced operator	0.939 (0.691, 1.277)	0.688		
Pre-treatment agent	1.346 (0.950, 1.908)	0.094		
Sedatives	2.398 (1.248, 4.606)	0.009	2.377 (1.224, 4.616)	0.011
Paralytic agents	0.476 (0.316, 0.717)	< 0.001	0.449 (0.293, 0.687)	< 0.001
Cormack–Lehane grade	1.168 (0.923, 1.477)	0.195		
No. of intubation attempts	1.460 (1.237, 1.723)	< 0.001	1.535 (1.291, 1.825)	< 0.001
Intubation duration	1.067 (1.025, 1.111)	0.002		

*OR* odds ratio, *CI* confidence interval

**Table 7:** Subgroup analysis

Variable	Total *n* = 958	First-attempt success	*p*	Intubation-related complications	*p*
Inexperienced operator, *n* (%)	639 (66.7)	398 (62.3)	< 0.001	170 (26.6)	0.785
Direct laryngoscopy	344 (53.8)	178 (51.7)		90 (26.2)	
Video laryngoscopy	295 (46.2)	220 (74.6)		80 (27.1)	
Experienced operator, *n* (%)	319 (33.3)	266 (83.4)	0.068	81 (25.4)	0.470
Direct laryngoscopy	121 (37.9)	95 (78.5)		28 (23.1)	
Video laryngoscopy	198 (62.1)	171 (86.4)		53 (26.8)	
Without predicted difficult airway, *n* (%)	764 (79.7)	563 (73.7)	< 0.001	192 (25.1)	0.871
Direct laryngoscopy	406 (53.1)	256 (63.1)		103 (25.4)	
Video laryngoscopy	358 (46.9)	307 (85.8)		89 (24.9)	
Video laryngoscopy group	493 (51.5)	391 (79.3)	0.223	133 (27.0)	0.056
Paralytics	73 (14.8)	54 (74.0)		13 (17.8)	
No paralytics	420 (85.2)	337 (80.2)		120 (28.6)	

In the power analysis referent to previous studies, we achieved a statistical power of 99–100% for our study populations (493 in the video laryngoscopy group and 465 in the direct laryngoscopy group). Therefore, our study has an adequate sample size and appropriate power. Hosmer–Lemeshow goodness-of-fit testing revealed that multivariate models were well fitted (*χ*^2^ = 6.419, *p* = 0.600 for first-attempt intubation success; *χ*^2^ = 1.666, *p* = 0.948 for intubation-related complications, respectively).

## Discussion

To our knowledge, this is the first study of the efficacy of video laryngoscopy when performed by non-anaesthesiologists in a general ward setting. The results of this study showed a higher first-attempt intubation success rate with video laryngoscopy than with direct laryngoscopy. However, there was no significant association of video laryngoscopy with lower intubation-related complications. Additionally, the number of intubation attempts was significantly lower in the video laryngoscopy group. Video laryngoscopy was also associated with improved visualisation of the glottis when compared with direct laryngoscopy.

In the general ward, tracheal intubation tends to be performed by less experienced operators and without capnography. Several studies have demonstrated that residents conducted 76–83% of first intubation attempts in critically ill patients [12, 19]. Bowles et al. reported that capnography was used in only 20% of general ward intubations [13]. In our hospital, all general ward tracheal intubations performed by inexperienced operators were supervised, involved capnography, and featured assistance by highly trained MET nurses. Accordingly, our study observed an overall first-attempt success rate of 69%, which is comparable to the rates of 56–75% reported for the ICU and emergency department (ED) [7, 9, 12, 20]. Furthermore, intubation-related complications were observed in 26% of the patients, and this was lower than the rates of 28–39% in the ICU and ED [1–3, 20–22].

Previous studies have reported an association of video laryngoscopy with an improved first-attempt success rate for difficult intubation cases; [10] in the ICU [7, 8, 18, 23, 24], ED [25, 26], and pre-hospital settings; [27] and in cases of in-hospital cardiac arrest [28]. However, recent randomised clinical trials (RCTs) [12, 20, 29] in ICU settings found that video laryngoscopy did not yield higher first-attempt tracheal intubation success rate than direct laryngoscopy. The MACMAN trial [12] emphasised that improved glottis visualisation with video laryngoscopy did not translate into an improved first-attempt success rate. The authors suggested that tracheal catheterisation under indirect vision was more difficult. However, our analysis suggests that first-attempt intubation success depends on complex interactions of the mode of laryngoscopy, level of operator experience, and prediction of difficult airways. In the MACMAN trial [12], 83% of tracheal intubations were performed by non-expert operators. Although another RCT by Janz et al. [20]. included tracheal intubations conducted by pulmonary and critical care medicine fellows, the fellows had each performed fewer than 5 tracheal intubations with video laryngoscopy during the study period and had a previous experience of only 10 tracheal intubations using video laryngoscopy. Also, in a single-centre pilot RCT by Griesdale et al. [29], video laryngoscopy did not yield an improved first-attempt success rate. However, that study included only 40 tracheal intubations conducted by novices (e.g. medical students or non-anaesthesiology residents), with an overall first-attempt success rate of 38%. Although there is an argument to use of video laryngoscopy could shorten the learning curve, a recent study of the GlideScope device demonstrated that 76 attempts would be required to master intubation whereas traditional cut-off for direct laryngoscopy is set at 50 attempts [30]. In other words, these RCTs were limited by the lack of sufficient time for the operators to acquire a reliable level of experience with video laryngoscopy.

There is a concern of video laryngoscopy is associated with severe complications. The MACMAN trial demonstrated that video laryngoscopy was associated with severe life-threatening complications and higher incidence of severe desaturation [12]. The results of our study did not show that video laryngoscopy reduced intubation-related complications, and video laryngoscopy was associated with more severe desaturation than direct laryngoscopy. However in our study, the incidence rates of a pre-intubation SpO_2_ < 80% and predicted difficult airway were higher in the video laryngoscopy group. This may affect the rate of intubation-related complications (Additional file 3: Table S3).

In this study, hospital mortality was higher in the video laryngoscopy group than in the direct laryngoscopy group. However, meta-analysis of the 12 RCTs showed that in-hospital mortality was not significantly different between video laryngoscopy and direct laryngoscopy [31]. We think many factors such as disease severity, organ dysfunction, underlying disease and comorbidities also contribute to mortality.

There were similar complications associated with the video laryngoscope such as the insertion of a styletted endotracheal tube through the right palatopharyngeal arch [32, 33]. In addition, even small amounts of oropharyngeal blood or vomitus can easily contaminated the lens of the video laryngoscope [34]. The GlideScope with a hyper-angulated blade is not the best device to learn intubation skills and required stylet mandatory. We cannot guarantee whether other types of video laryngoscopes would yield similar results.

This study had several limitations. First, this was a retrospective analysis, and the operators chose the laryngoscopy mode according to their preference. Thus, the device used for the first intubation attempt was not randomly assigned. Accordingly, the level of operator experience may have influence the device success rates. However, we performed an adjustment for differences in baseline characteristics of patients using a propensity-score matching to reduce the effect of confounding factors. As we described in Additional file 2: Tables S2 and Additional file 4: Table S4, the results of the propensity-score matching showed that the use of video laryngoscopy is associated with increased odds of successful intubation on first attempt compared with the direct laryngoscopy. Second, we did not demonstrate the Mallampati score and the percentage of glottis opening (POGO) score. Unfortunately, these variables were not routinely recorded in our intubation data collection sheet. Third, the experienced operators in our study may differ from those in other studies [7, 12, 20]. In previous studies, the authors defined an expert operator as a critical care medicine attending physician or anaesthesiologist. However, it is unlikely that every tracheal intubation in real-world general ward settings is performed under the supervision of such experts. In our hospital, an anaesthesiologist is not always available for tracheal intubation outside of the operating room. A rescue airway team, which includes an anaesthesiologist, is activated only in ‘cannot intubate, cannot oxygenate’ situations. In addition, the results of our analysis showed that the first-attempt success rate of experienced operators was sufficiently high to allow them to supervise inexperienced operators. Therefore, stratification of the level of operator experience according to board certification seems appropriate. Fourth, only 21% of patients received paralytics, which is associated with improved first-attempt success, improved Cormack–Lehane grade, and decreased procedure-related complications in critically ill patients [35]. Low rate of neuromuscular blockade use could have decreased the first-attempt success rate, and the video laryngoscopy group seems to be an awake intubation group which require expertise. However, in this study the first-intubation success rate was lower in direct laryngoscopy group, which usage of neuromuscular blockers was higher. In addition, after propensity-score matching, the first-attempt success rate was greater in video laryngoscopy group than direct laryngoscopy group. Therefore, further study is needed for this issue.

A strength of our study is the enrolment of a relatively large number of general ward patients who underwent tracheal intubation; accordingly, the results of our study will provide information to support the selection of intubation devices in a general ward setting.

## Conclusion

In this study, we observed a higher first-attempt intubation success rate with video laryngoscopy than with direct laryngoscopy in a general ward setting. However, video laryngoscopy did not reduce intubation-related complications. Furthers trials on best way to perform intubation in the emergency settings are required.

## Additional files

**Additional file 1: Table S1.** Characteristics of patients after propensity-score matching.

**Additional file 2: Table S2.** Outcome characteristics.

**Additional file 3: Table S3.** Factors associated with severe life-threatening complications.

**Additional file 4: Table S4.** Analysis of outcomes.

## Abbreviations

ICU: intensive care unit
MET: medical emergency team
ED: emergency department
RCT: randomised clinical trial
