# Supplementary material for: Video laryngoscopy versus direct laryngoscopy for first-attempt tracheal intubation in the general ward
Source: Ann Intensive Care. 2018 Aug 13;8:83. doi: 10.1186/s13613-018-0428-0 (PMC6089856; doi:10.1186/s13613-018-0428-0)
Supplement: Supplementary file 1 — Additional file 1: Table S1. Characteristics of patients after propensity-score matching. [file 13613_2018_428_MOESM1_ESM.docx]

**Table S1. Characteristics of patients after propensity score matching**

| Variable | Total  *n* = 600 | Direct laryngoscopy  *n* = 300 | Video laryngoscopy  *n* = 300 | SDM |
| --- | --- | --- | --- | --- |
| Age (years) | 63.1 ± 1.9 | 62.8 ± 13.1 | 63.5 ± 12.6 | 0.060 |
| Male, *n* (%) | 398 (66.3) | 199 (66.3) | 199 (66.3) | 0.000 |
| Medical department, *n* (%) | 480 (80.0) | 238 (79.3) | 242 (80.7) | 0.033 |
| Systolic blood pressure (mm Hg) | 124 ± 33 | 125 ± 33 | 123 ± 34 | 0.050 |
| Diastolic blood pressure (mm Hg) | 72 ± 23 | 73 ± 21 | 71 ± 24 | 0.084 |
| Heart rate (beats per minute) | 121 ± 27 | 121 ± 27 | 120 ± 28 | 0.048 |
| Oxygen saturation (%) | 94 (88, 98) | 94 (88, 98) | 94 (89, 98) | 0.093 |
| Severe desaturation (SpO_2_ <80%), *n* (%) | 50 (8.3) | 28 (9.3) | 22 (7.3) | 0.07 |
| Predicted difficult airway, *n* (%) | 112 (18.7) | 54 (18.0) | 58 (19.3) | 0.034 |
| Experienced operator *n*, (%) | 175 (29.2) | 88 (29.3) | 87 (29.0) | 0.007 |
| Pre-treatment agent, *n* (%) | 470 (78.3) | 234 (78.0) | 236 (78.7) | 0.016 |
| Sedatives, *n* (%) | 556 (92.7) | 279 (93.0) | 277 (92.3) | 0.026 |
| Paralytic agents, *n* (%) | 106 (17.7) | 53 (17.7) | 53 (17.7) | 0.000 |

*SDM* Standardized difference of means
