# Supplementary material for: Video laryngoscopy versus direct laryngoscopy for first-attempt tracheal intubation in the general ward
Source: Ann Intensive Care. 2018 Aug 13;8:83. doi: 10.1186/s13613-018-0428-0 (PMC6089856; doi:10.1186/s13613-018-0428-0)
Supplement: Supplementary file 2 — Additional file 2: Table S2. Outcome characteristics. [file 13613_2018_428_MOESM2_ESM.docx]

**Table S2. Outcome characteristics**

|  | Before PS matching | | | After PS matching | | |
| --- | --- | --- | --- | --- | --- | --- |
| Variables | Total (*n*=958) | Direct laryngoscopy (*n*=465) | Video laryngoscopy (*n*=493) | Total (*n*=600) | Direct laryngoscopy (*n*=300) | Video laryngoscopy (*n*=300) |
| First-attempt success | 664 (69.3) | 273 (58.7) | 391 (79.3) | 420 (70.0) | 181 (60.3) | 239 (79.7) |
| Complications | 251 (26.2) | 118 (25.4) | 133 (27.0) | 157 (26.0) | 80 (26.7) | 77 (25.7) |

*PS* propensity-score
