# Supplementary material for: Video laryngoscopy versus direct laryngoscopy for first-attempt tracheal intubation in the general ward
Source: Ann Intensive Care. 2018 Aug 13;8:83. doi: 10.1186/s13613-018-0428-0 (PMC6089856; doi:10.1186/s13613-018-0428-0)
Supplement: Supplementary file 3 — Additional file 3: Table S3. Factors associated with severe life-threatening complications. [file 13613_2018_428_MOESM3_ESM.docx]

**Supplementary Table S3. Factors associated with severe life-threatening complications**

| Variable | Univariate analysis | *p* | Multivariate analysis | *p* |
| --- | --- | --- | --- | --- |
|  | OR (95% CI) |  | OR (95% CI) |  |
| Age | 0.997 (0.980, 1.013) | 0.692 |  |  |
| Female | 0.822 (0.498, 1.357) | 0.443 |  |  |
| Video laryngoscopy | 1.441 (0.897, 2.315) | 0.131 |  |  |
| Medical department | 1.264 (0.758, 2.108) | 0.368 |  |  |
| Systolic blood pressure (mm Hg) | 0.986 (0.979, 0.994) | <0.001 | 0.987 (0.979, 0.995) | 0.002 |
| Diastolic blood pressure (mm Hg) | 0.985 (0.973, 0.996) | 0.009 |  |  |
| Heart rate (beats per minute) | 0.997 (0.988, 1.005) | 0.449 |  |  |
| Oxygen saturation (%) | 0.968 (0.951, 0.984) | <0.001 |  |  |
| Severe desaturation (SpO2 <80%), n (%) | 4.250 (2.278, 7.931) | <0.001 | 4.339 (2.262, 8.324) | <0.001 |
| Predicted difficult airway | 1.036 (0.583, 1.840) | 0.904 |  |  |
| Experienced operator | 1.089 (0.668, 1.776) | 0.732 |  |  |
| Pre-treatment agent | 0.700 (0.422, 1.161) | 0.167 |  |  |
| Sedatives | 0.779 (0.361, 1.683) | 0.526 |  |  |
| Paralytic agents | 0.923 (0.513, 1.660) | 0.788 |  |  |
| Cormack–Lehane grade | 0.863 (0.562, 1.325) | 0.502 |  |  |
| No. of intubation attempts | 1.008 (0.760, 1.336) | 0.959 |  |  |
| Intubation duration | 1.000 (0.933, 1.071) | 0.989 |  |  |

*OR* odds ratio, *CI* confidence interval

Severe life-threatening complications were death, cardiac arrest, hypotension and severe desaturation.[17]
