# Supplementary material for: Video laryngoscopy versus direct laryngoscopy for first-attempt tracheal intubation in the general ward
Source: Ann Intensive Care. 2018 Aug 13;8:83. doi: 10.1186/s13613-018-0428-0 (PMC6089856; doi:10.1186/s13613-018-0428-0)
Supplement: Supplementary file 4 — Additional file 4: Table S4. Analysis of outcomes. [file 13613_2018_428_MOESM4_ESM.docx]

**Table S4. Analysis of outcomes**

| Variables | Model | *n* | OR (95% CI) | *P* |
| --- | --- | --- | --- | --- |
| First-attempt success | Univariate analysis | 958 | 2.696 (2.026, 3.587) | <0.001 |
|  | Multivariable analysis | 958 | 3.058 (2.192, 4.267) | <0.001 |
|  | Propensity score | 600 | 2.450 (1.696, 3.539) | <0.001 |
| Complications | Univariate analysis | 958 | 1.086 (0.814, 1.450) | 0.573 |
|  | Multivariable analysis | 958 | 1.173 (0.865, 1.591) | 0.306 |
|  | Propensity score | 600 | 0.942 (0.638, 1.392) | 0.765 |

*OR* odds ratio, *CI* confidence interval

Odds Ratio is for the video laryngoscopy group as compared with the direct laryngoscopy group

Univariate analysis: Logistic regression model

Multivariable analysis: Logistic regression model with backward elimination method

Propensity score matching analysis: Conditional Logistic regression model for matched pairs data
